# Associations between symptom and neurocognitive dimensions in clinical high risk for psychosis

**DOI:** 10.1016/j.scog.2022.100260

**Published:** 2022-06-02

**Authors:** Ingvild Aase, Johannes H. Langeveld, Inge Joa, Jan Olav Johannessen, Ingvild Dalen, Wenche ten Velden Hegelstad

**Affiliations:** a TIPS Centre for Clinical Research in Psychosis, Clinic for Adult Mental Health Care, Stavanger University Hospital, P.O. 8100, 4068 Stavanger, Norway; b Faculty of Health Sciences, University of Stavanger, 4036 Stavanger, Norway; c Research Department, Stavanger University Hospital, P.O. 8100, 4068 Stavanger, Norway; d Faculty of social sciences, Stavanger University Hospital, 4036 Stavanger, Norway

**Keywords:** Clinical high risk for psychosis (CHR), Structural interview for prodromal syndromes (SIPS), Positive symptoms, Negative symptoms, Disorganization symptoms, Cognition

## Abstract

**Introduction:**

Clinical high risk for psychosis (CHR) is associated with mild cognitive impairments. Symptoms are clustered into positive, negative and disorganization symptoms. The association between specific symptom dimensions and cognitive functions remains unclear. The aim of this study was to investigate the associations between cognitive functions and positive, negative, and disorganization symptoms.

**Method:**

53 CHR subjects fulfilling criteria for attenuated psychotic syndrome in the Structural Interview for Prodromal Syndromes (SIPS) were assessed for cognitive function. Five cognitive domain z-scores were defined by contrasting with observed scores of a group of healthy controls (*n* = 40). Principal Components Analyses were performed to construct general cognitive composite scores; one using all subtests and one using the cognitive domains. Associations between cognitive functions and symptoms are presented as Spearman's rank correlations and partial Spearman's rank correlations adjusted for age and gender.

**Results:**

Positive symptoms were negatively associated with executive functions and verbal memory, and disorganization symptoms with poorer verbal fluency. Negative symptoms were associated with better executive functioning. There were no significant associations between the general cognitive composites and any of the symptom domains, except for a trend for positive symptoms.

**Conclusion:**

In line with previous research, data indicated associations between positive symptoms and poorer executive functioning. Negative symptoms may not be related to executive functions in CHR the same way as in psychosis. Our results could indicate that attenuated positive symptoms are more related to cognitive deficits in CHR than positive symptoms in schizophrenia and FEP.

## 1 Introduction

Deficits in a variety of cognitive functions are common in all stages of psychosis (Addington et al., 2003; Barder et al., 2013; Friis et al., 2002; Kurtz, 2005; Palmer et al., 2009; Schaefer et al., 2013). The term clinical high risk for psychosis (CHR) (McGorry et al., 2003; Yung and McGorry, 1996) refers to subthreshold psychotic symptoms and is also associated with cognitive impairments (Bora et al., 2014; Fusar-Poli et al., 2012; Giuliano et al., 2012; Hawkins et al., 2004; Pukrop and Klosterkötter, 2010). Cognitive impairments may lie at an intermediate level between those in psychosis and those in healthy controls (Brewer et al., 2006; Eastvold et al., 2007; Hawkins et al., 2004; Kim et al., 2011). Consequently, it has been proposed that they may play a mediational role in a trajectory from CHR to psychosis. (Bora and Murray, 2013; Fuller et al., 2002; Lam et al., 2018).

In schizophrenia, positive symptoms are associated with deficits in verbal memory (Brébion et al., 2006; Bruder et al., 2004; Heinrichs and Zakzanis, 1998) executive functions (Freedman and Brown, 2011; Guillem et al., 2008; Mcgurk et al., 1997; Sabhesan and Parthasarathy, 2005), verbal fluency (Henry and Crawford, 2005) and verbal working memory (Bruder et al., 2011). Negative symptoms are associated with deficits in executive functions (Dibben et al., 2009; Nieuwenstein et al., 2001), verbal fluency, verbal memory and learning, IQ (de Gracia Dominguez et al., 2009) and attention (Sanz et al., 2012). In a meta-analysis by de Gracia Dominguez et al. (2009) disorganization symptoms were associated with impairments in attention/vigilance, visual learning and memory. A study by Cuesta and Peralta (1995) demonstrated disorganization symptoms to be associated with deficits in language functions and verbal memory. Finally, disorganization symptoms may be associated with a decline in general intellectual functioning and attention span (Basso et al., 1998).

In first episode psychosis (FEP) studies by our group, positive symptoms were associated with slower motor speed (Rund et al., 2004) and auditory hallucinations appeared related to deficits in verbal working memory (Gisselgård et al., 2014). Lindsberg et al. (2009) found negative symptoms in FEP to be associated with more general cognitive impairments, while disorganization symptoms were related to more specific deficits in general intelligence, memory, executive functions, sustained attention and sensorimotor function. However, no associations between positive symptoms and cognition were found. Negative symptoms in FEP subjects have been linked to impairments in verbal memory (Hovington et al., 2013).

According to Ohmuro et al. (2015), this picture appears no less complicated in CHR. Some of the attenuated symptoms may reflect underlying “core” psychotic processes, and other symptoms may represent epiphenomena associated with other, non-psychotic clinical conditions (“clinical noise”). Frommann et al. (2011) divided a group of CHR subjects (*n* = 205) into a late prodromal state (LPS) and early prodromal state (EPS) and found executive dysfunctions to be present only in the LPS group*.* One study concerning individuals with attenuated positive symptoms in a non-clinical population found an association between positive symptoms and deficits in executive functions (Martín-Santiago et al., 2016). Similarly, a study by a group connected to ours, found working memory to be associated with attenuated positive symptoms (Anda et al., 2019) Negative symptoms in CHR appear associated with a reduction in verbal fluency (Shin et al., 2016; Vargas et al., 2018). Leanza et al. (2018) found negative symptoms to be related to poorer nonverbal intelligence scores*.* Negative symptoms have been found to be a mediator between neurocognitive deficits and daily life functional outcome in CHR (Glenthøj et al., 2017; Meyer et al., 2014). The previously mentioned study by Vargas et al. (2018) also indicated an association between disorganization symptoms and reduced verbal fluency. Several studies in CHR have demonstrated global working memory, verbal fluency, attention and memory impairments (Sheffield et al., 2018). A meta-analysis found mild impairments in most cognitive areas: Executive functions, general intelligence, verbal- and visual memory, verbal fluency, working memory, attention and social cognition (Fusar-Poli et al., 2012). Other studies have found impairments in executive functions (Morey et al., 2005; Schultze-Lutter et al., 2007; Vargas et al., 2018). In a previous study at our site, individuals at CHR demonstrated impairments in inhibition (Aase et al., 2018). However, Brewer et al. (2006) addresses a caveat in a review of cognitive performance in CHR cohorts by proposing that cognitive deficits in psychotic disorders may fluctuate in relation to psychopathology and stage of illness and that this would limit the generalizability when comparing findings across different illness stages.

The differentiation of cognition in relation to psychopathology into individual tests or functions is however cause of some debate. Neuropsychology has a long tradition to group tests into cognitive domains (Lezak, 2004) . However, in a recent review, researchers were encouraged to apply a more unitary view of cognition when investigating associations between cognition and psychiatric illness, and aimed to design one overall general cognitive composite score described as “the C factor” (Abramovitch et al., 2021). A general cognitive composite score has been computed in neurocognitive studies using Principal Component Analysis (PCA) in psychotic disorders (Harvey et al., 2016; Harvey et al., 2014; Keefe et al., 2006) and in CHR (Barbato et al., 2013). In the baseline data of neurocognitive deficits in the CATIE Schizophrenia trial (*n* = 1460) by Keefe et al. (2006), a composite score was calculated by principal component and confirmatory factor analyses. These analyses suggest that a single dimension of cognition, or a single composite score, was the best fit, or best representation of the data. According to Harvey et al. (2016), cognition is best understood as a single latent trait, and this single measure may enhance the robustness of further analysis. In a review of cognitive deficits in psychotic disorders, a generalized cognitive deficit factor was associated with poorer functional outcomes in all groups from CHR to schizophrenia (Sheffield et al., 2018). In the Keefe et al. (2006) study, no statistically significant correlations were found between neither the composite score nor the cognitive domains and positive- and negative symptom domains. However, the conclusion was clear, and it was suggested that a single component of cognition best described the data, indicating cognition to be best understood as a single latent trait.

The three symptom dimensions positive, negative and disorganization symptoms are however important in understanding the CHR state, as they are represented in two of the most widely used interviews defining CHR, the SIPS - and the CAARMS interview (Mam-Lam-Fook et al., 2017). Previously, we published a paper in Schizophrenia Research Cognition (Aase et al., 2021) where we analyzed how cognitive functions were associated with positive symptoms longitudinally over a two-year follow-up period. However, overall the literature on symptom-specific cognitive deficits in CHR is relatively scarce compared to in FEP and schizophrenia. Findings across CHR studies regarding symptomatology and cognition are also divergent.

In the present paper, disorganization and negative symptoms are included in addition to positive symptoms, and we investigate associations between cognition and clinical symptomatology at baseline with theory-driven cognitive domains and by computing a general cognitive composite score. Comparing these approaches can shed light on the question whether individual cognitive functions on the one, or a general composite cognition score on the other hand, best fit the symptom dimensions in CHR.

To our knowledge, few if any other studies have investigated the positive, negative and disorganization symptom dimensions simultaneously to compare associated cognitive impairments. This enables a direct comparison regarding the symptom domains and load on cognitive deficits.

The aim of this study was thus to investigate the associations of positive, negative, and disorganization symptoms with the cognitive functioning domains of attention, verbal memory, verbal fluency, executive function, and general intelligence, as well as with cognitive composite scores representing cognition as a single latent trait.

## 2 Methods

### 2.1 Participants and study design

The present study is part of the overall Norwegian Prevention (POP) study conducted at TIPS, early Norwegian detection of psychosis site. The POP study is described elsewhere (Joa et al., 2021; Joa et al., 2015; Aase et al., 2021). 53 CHR individuals and 40 non help-seeking healthy controls, matched on age, gender and cultural background, were recruited between 2012 and 2017 from the ongoing Prevention of Psychosis (POP) study in a Norwegian early detection of psychosis site, TIPS (Joa et al., 2021; Joa et al., 2015; Aase et al., 2021). The present study includes a subset of the main study consisting of participants who had completed neuropsychological assessments (*n* = 53). Inclusion criteria were: Rogaland County resident; age 13–65 years; fulfilling the diagnostic criteria for one of three psychosis risk syndromes: Lifetime Attenuated Positive Symptom Syndrome (APSS); (2) lifetime “Brief Limited Intermittent Psychotic Symptoms” (BLIPS) or (3) “Lifetime Genetic Risk and Deterioration syndrome (GDR) as defined using the Structural Interview for Prodromal Syndromes (SIPS) interview (Miller et al., 2003); no current or lifetime psychotic episode; the symptoms are not better explained by axis 1, axis 2 (in the DSM IV) or substance abuse disorder; no antipsychotic medication for more than 4 weeks lifetime, regardless of dosage; no known neurological or endocrine disorder; no mental retardation; sufficient mastery of a Scandinavian language; able to understand and sign an informed consent form (parents/legal guardians gave informed consent for subjects under 16 years of age. Healthy controls had no past or present mental disorder, no known family history of mental disorder in first degree relatives, no past or current drug abuse. Further inclusion criteria and demographics for the healthy controls are described elsewhere (Aase et al., 2018).

### 2.2 Clinical measures

Symptoms were assessed using the (SIPS)-interview (Miller et al., 2003; Miller et al., 1999), version 5.0 (McGlashan et al., 2012). All items in SIPS are divided into positive, negative, disorganization, and general symptoms and are ranged in severity from 0 to 6. SIPS cutoff for inclusion was a score of “3 = moderate”, “4= moderate severe”, or “5 =severe, but not psychotic” on a at least one of the positive symptom items (P1 Unusual thought content/delusional ideas, P2 Suspiciousness/persecutory ideas, P3 Grandiose Ideas, P4 Perceptual abnormalities/hallucinations, P5 Disorganized communication). A score of 6 is described as “severe and psychotic” and serves as an exclusion criterion, persons scoring 6 are referred to our FEP program. Extensively trained psychiatric nurses supervised by clinical psychologists or psychiatrists conducted the SIPS interviews.

Clinical psychologists or psychiatrists conducted diagnostic interviews using the Structured Clinical Interview for DSM-IV Axis I Disorders (SCID-I) (First et al., 1994). The interviews were presented at weekly staff meetings. These meetings aimed at reaching a consensus regarding the fulfillment of inclusion criteria and diagnoses. Reliability on SCID-I for this team is good at kappa =0.9 (Weibell et al., 2013).

The Global Assessment of Functioning (GAF-M) scale (Hall, 1995) is included in the SIPS interview. GAF-M scores comprehend both function and symptoms. The range of GAF-M is 0–100 where 0 represents the poorest level of function.

### 2.3 Neuropsychological measures and functional domains

Clinical psychologists or psychiatric nurses with specialized training administrated neuropsychological testing. To provide a general organizational framework, tests were arranged into five functional domains: Attention, verbal memory, verbal fluency, executive functions and general intelligence. This was guided by the grouping of tests as presented in a meta-analysis of cognition in first-episode schizophrenia (Mesholam-Gately et al., 2009) as well as in a meta-analysis of cognition in CHR (Giuliano et al., 2012). For these domains, we used acknowledged tests from Delis-Kaplan Executive Function System (D-KEFS), (Delis et al., 2001), Wechsler Adult Intelligence Scale (WAIS III) (Wechsler, 1997), Wechsler Memory Scale (WMS-III) (Wechsler, 1997), Trail Making Test (War Department Adjutant General's Office, 1944) and California Verbal Learning Test (CVLT) (Delis, 2000) Table 1 presents details of the tests. Furthermore, following the approaches of the CATIE Schizophrenia trial (*n* = 1493) by Keefe et al. (2006) and Veteran Affairs Cooperative Studies Program study #572 (SZ *n* = 3942, BPI *n* = 5414) by Harvey et al. (2016), composite scores were estimated based on 1) all of the 15 individual tests and 2) the five domains.

**Table 1:** Functional cognitive domains and tests with test variable employed.

Cognitive domain	Name of test	Variable employed
Attention	D-KEFS CWIT Color Naming*	Time to completion (seconds)
	D-KEFS CWIT Word Reading*	Time to completion (seconds)
	WAIS-III Digit Span Forward	No. of correctly reported digits
Verbal Memory	CVLT-II List A Total Recall	No. of correct words reported from list A in five trials
	CVLT-II Short-Delay Free Recall: List A	No. of correctly reported words from list A
Verbal fluency	D-KEFS VFT Letter Fluency	No. of correctly reported words (F, A, S)
	D-KEFS VFT Category Fluency (animals)	No. of correctly reported animals
	D-KEFS VFT Category Fluency (names)	No. of correctly reported boys names
	D-KEFS VFT Category Switching	No. of correct shifts between categories (fruit, furniture)
Executive functions	WAIS-III Digit Span Backward *(Working Memory)*	No. of correctly reported digits
	D-KEFS CWIT Inhibition* *(Inhibition)*	Time to completion (seconds)
	TMT-B* (*Cognitive Flexibility)*	Time to completion (seconds)
	D-KEFS CWIT Inhibition/Switching* (Cognitive flexibility)	Time to completion (seconds)
General intelligence	WAIS-III Vocabulary	Accuracy of words defined
	WAIS-III Block Design	No. of correctly produced blocks within time limit

Notes: All variables employed are raw scores. A higher score on the tests indicates a better performance, unless tests are marked with an asterix (*) in which a higher score indicates poorer performance. D-KEFS CWIT = Delis-Kaplan Executive Function System Color Word Interference Test (“Stroop”), D-KEFS VFT = Delis-Kaplan Executive Function System Verbal Fluency Test (Delis et al., 2001), WAIS-III = Wechsler Adult Intellegence Scale (Wechsler, 1997), WMS-III = TMT = Trail Making Test (War Department Adjutant General's Office, 1944), CVLT-II = California Verbal Learning Test (Delis, 2000).

### 2.4 Data analyses

Neuropsychological test scores of healthy controls (*n* = 40) were included for computing test z-scores, for CHR subjects by subtracting the mean of the scores of the healthy control group and then dividing by the sample standard deviation of the same group. Cognitive domain scores in CHR were further defined as mean z-scores of included tests, regarding z-scores go to (Aase et al., 2021). Composite scores were estimated using principal component analysis (PCA) on correlation matrices of 1) the 15 individual tests or 2) the five domains, where the resulting composites explained respectively 29 and 46% of the total variance of the standardized test scores/domains. Variable loadings for each of the composites are presented in Supplementary Tables S1 and S2. Both composites were used in the analyses.

Inspecting boxplots and QQ plot for the variables revealed that most of the variables did not follow a normal distribution. Hence, we present descriptive statistics as medians and interquartile ranges (IQR). For the same reason, we present associations between cognitive functioning and symptoms as Spearman's rho rank correlations and partial rho correlations adjusted for age and gender. Estimates of correlations are presented with 95% confidence intervals (CI) and *p*-values from tests of no association.

Descriptive statistics were performed in IBM SPSS Statistics v. 24 (Spss, 2016) and PCA and correlation analysis was performed in R, v. 3.4. PCA was performed using function prcomp from the Stats package. Spearman's rho was estimated using function Spearman Rho in package Desk Tools and tested using function cor. Test of the Stats package, partial Spearman was estimated and tested using function partial Spearman in package PResiduals (Liu et al., 2018).

## 3 Results

### 3.1 Demographics and clinical characteristics

Table 2 presents demographics and clinical characteristics of the sample (*n* = 53). Participants were mostly adolescents between 15 and 19 years of age (median 17, range 13–39). The proportion of female participants was nearly 60%. Fifty of the subjects were born in Scandinavia and three of the subjects were born in other European countries. Negative and disorganization symptoms were significantly correlated (rho, =0.503, *p* < 0.001), and negative symptoms were also associated with general symptoms (rho =0.63, *p* = 0.0001).

**Table 2:** Demographic and clinical data for 53 Clinical High Risk (CHR) subjects at baseline.

Characteristics	All (*n* = 53)
Age	17 (15–19)
Gender (counts female/male)	31/22
GAF-M at baseline	47 (40–55)
Cultural background (counts Nordic/other European)	50/3
SIPS positive symptoms at baseline	
Sum scores	10 (8–13)
Mean scores	2.0 (1.6–2.6)
SIPS disorganization symptoms at baseline	
Sum scores	3 (1–4)
Mean scores	0.8 (0.3–1.0)
SIPS negative symptoms at baseline	
Sum scores	11 (6–17)
Mean scores	1.8 (1.0–2.8)

Notes: All data are presented as median (inter quartile range) unless otherwise stated.

GAF M = Global Assessment of Functioning. SIPS=Structured Interview for Psychosis-risk Syndromes.

GAF-M scores (Hall, 1995) were at the lower end of the scale (median 46, IQR 40 to 55). A GAF-M score from 44 to 47 indicates impaired in two of the following areas; work/school, confronting behavior, relationships with family, relationship with friends, or having moderate to severe symptoms. For a description of the z-scores go to Table 3.

**Table 3:** Overview of cognitive domain z-scores at baseline for the 53 CHR subjects.

Cognitive domain	n	median (IQR)	min, max
Attention	50	−0.40 (−1.28, 0.19)	−2.43, 1.27
Verbal Memory	51	−0.26 (−1.24, 0.48)	−2.76, 1.76
Verbal fluency	52	−0.44 (−0.98, −0.05)	−2.26, 1.50
Executive function	51	−0.21 (−1.05, 0.26)	−2.24, 1.22
General intelligence	53	−0.46 (−1.15, 0.21)	−2.59, 1.49

Notes: Z scores for individual tests were computed by standardising against an internal group of healthy controls (n = 40). Cognitive domain z scores were defined as the mean of the z-scores of the included tests. Descriptive statistics of the domain z-scores are presented here as median, interquartile range (as 25th and 75th percentile) and full range (as minimun and maximum scores).

Close to half of the participants fulfilled criteria for affective disorder. Two of these fulfilled criteria for Bipolar II disorder, and none fulfilled criteria for Bipolar 1 disorder (Table 4). Anxiety disorders were the second most common (9 of 52, i.e. 17%), and two of the subjects were diagnosed with a substance abuse disorder. Less than 15% of the participants (7 of 53 i.e. 13.5%) did not fulfill criteria for any full diagnosis.

**Table 4:** Main DSM-IV diagnoses (*n* = 53).

DSM-IV code	Diagnosis	
296.xx	Affective disorders (2/22 Bipolar II Disorder)	22
309.xx	Adjustment Disorders	5
300.xx	Anxiety Disorders	9
300.9	Unspecified Mental Disorder (nonpsychotic)	2
300.3	Obsessive Compulsive Disorder	2
307.8	Pain Disorder Associated with Psychological Factors	1
300.4	Dysthymia	1
304.8	Polysubstance Dependence	1
314.9	Attention-Deficit/Hyperactivity Disorder NOS	1
292.90	Cannabis related Disorder NOS	1
799.9	Diagnosis deferred	7
999.99	Missing	1

Note Main DSM-IV diagnosis for the 53 CHR sub.

### 3.2 Cognitive functioning as related to positive-, negative-, and disorganization symptoms

The Spearman's rho (*p*) and partial Spearman's rho (*ρ*_p_) correlation coefficients adjusted for age and gender between positive-, disorganization-, and negative symptoms and scores on neuropsychological tests are presented in Table 5*.* Most of the statistically significant correlations (*p* ≤ 0.05) were of moderate or small, close to moderate size (Cohen, 1988) (between 0.28 and 0.34 in absolute values), and the confidence intervals were generally wide. Statistically significant adjusted correlations were seen for the following associations: Higher levels of negative symptoms were associated with better performance on executive functions (*ρ*_p_ 0.33, 95% CI 0.08 to 0.54) and for the disorganization symptoms domain, there was an association with poorer verbal fluency (*ρ*_p_ − 0.29, 95% CI -0.52 to −0.01). When not adjusted for age and gender, we found two statistically significant negative correlations between positive symptoms and executive functions (*ρ*-0.34, 95% CI -0.56 to 0.07) and verbal memory (*ρ*_−_-0.28, CI 95% (−0.52-, −0.01). For negative symptoms, also the unadjusted correlation between executive functions and negative symptoms was statistically significant (*ρ* 0.33, CI 95% (0.08–0.54). The composite scores were not statistically significantly associated with any of the symptom scores, however there was a trend for a negative association between positive symptoms and the composite based on the individual tests (*ρ*_p_ and *ρ* − 0.27, *p* = 0.063 and 0.065, respectively). Overall, adjustment for age and gender had only little effect on the estimated correlations.

**Table 5:** Subthreshold psychotic symptoms associated with cognitive domains and tests among 53 CHR subjects.

	Positive symptoms				Disorganized symptoms				Negative symptoms			
	*ρ* (95% CI)	*p*	*ρ*_p_ (95% CI)	*p*	*ρ* (95% CI)	*p*	*ρ*_p_ (95% CI)	*p*	*ρ* (95% CI)	*p*	*ρ*_p_ (95% CI)	*P*
*Composite test* *(n* *=* *48)*	−0.27 (−0.51, 0.02)	0.065	−0.27 (0.52–0.01)	0.063	−0.09 (−0.37, 0.19)	0.52	−0.14 (−0.41, 0.15)	0.35	0.06 (−0.23, 0.34)	0.70	0.07 (−0.23, 0.35)	0.66
*Composite domains* *(n* *=* *48)*	−0.24 (−0,49, 0.04)	0.097	−0.23 (−0.49, 0.04)	0.12	−0.09 (−0.36, 0.20)	0.54	−0.09 (−0.36–0.19)	0.54	0.02(−0.26–0.31)	0.87	0.04(−0.25-0.33)	0.78
*Attention* *(n* *=* *50)*	−0.07 (−0.34, 0.21)	0.64	−0.09 (−0.34, 0.17)	0.49	−0.17 (−0.43, 0.11)	0.24	−0.14 (−0.40, 0.14)	0.33	0.17 (−0.11, 0.43)	0.23	0.19 (−0.09, 0.44)	0.18
*Verbal Memory* *(n* *=* *51)*	**−0.28 (−0.52, −0.01)**	**0.046**	−0.27 (−0.50, 0.00)	0.052	0.04 (−0.24, 0.31)	0.77	0.04 (−0.24, 0.31)	0.77	0.17 (−0.11, 0.43)	0.22	0.17 (−0.14, 0.45)	0.28
*Verbal Fluency* *(n* *=* *52)*	−0.21 (−0.45, 0.07)	0.14	−0.27 (−0.53, 0.03)	0.077	−0.17 (−0.42, 0.11)	0.23	**−0.29 (−0.52, −0.01)**	**0.040**	−0.14 (−0.40, 0.13)	0.31	−0.17 (−0.43, 0.11)	0.23
*Executive Functions (n* *=* *51)*	**−0.34 (−0.56, −0.07)**	**0.015**	−0.30 (−0.55, 0.00)	0.053	0.15 (−0.13, 0.41)	0.30	0.14 (−0.13, 0.39)	0.31	**0.31 (0.04, 0.54)**	**0.028**	**0.33 (0.08, 0.54)**	**0.011**
*General intelligence (n* *=* *53)*	−0.01 (−0.28, 0.27)	0.97	−0.01 (−0.29, 0.27)	0.93	−0.02 (−0.29, 0.25)	0.88	0.10 (−0.18, 0.37)	0.47	0.04 (−0.23, 0.31)	0.75	0.10 (−0.18, 0.37)	0.49

Notes: Results given as Spearmans's rho correlations (p) and partial Spermans's rho correlations (p_p_) adjusted for age and gender, with following 95% confidence intervals (CI). Statistically significant *p*-values (< 0.05) are marked in **bold**.

## 4 Discussion

The main findings of this study were that executive functions were negatively associated with positive symptoms, but positively associated with negative symptoms in subjects at CHR for psychosis. Further, we found correlations between positive symptoms and impairments in verbal memory and between disorganization symptoms and deficits in verbal fluency.

There was a trend for an association between positive symptoms and general cognitive functioning.

Attenuated positive symptoms being correlated with executive functioning is in line with previous research on non-clinical populations (Martín-Santiago et al., 2016) and also with a previous study from our group (Aase et al., 2021). Here we linked the course of positive symptoms over two years with neuropsychological functioning and found that better executive function at baseline was associated with lower levels of positive symptoms at follow-up. The finding lends itself to some speculation as to underlying mechanisms or common factors. For instance: Executive functions enable a person to flexibly and quickly adapt to different circumstances. This concerns overt (motor) behavior as well as mental operations involving mental control, inhibiting task-irrelevant responses and focusing attention on relevant stimuli (Diamond, 2013). Following this line of reasoning, impaired executive control would lead a person to direct attention to more salient stimuli at the cost of less salient stimuli. In an earlier dichotic listening study by our group, CHR subjects demonstrated impairments in inhibition when instructed to report the least salient stimuli (Aase et al., 2018). Hallucinations and perceptual abnormalities arguably appear more salient compared to stimuli requiring attention in order to complete some executive task. Another relevant part of executive function concerns self-monitoring. Having a meta-level view on mental tasks and operations enables the person to self-correct when necessary. Both hallucinations and overvalued ideas can be viewed as problems steering attention away from internal stimuli. Along these lines of reasoning, executive problems could be viewed as underlying certain abnormal perceptual experiences, an idea not new to the field of psychosis (Hugdahl, 2009; Waters et al., 2012), but not extensively studied in CHR.

The association between positive symptoms and verbal memory is in line with findings from studies of cognitive function in established psychosis (Addington et al., 1991; Brébion et al., 2006; Green and Walker, 1985; Heinrichs and Zakzanis, 1998) and in first episode psychosis (Mesholam-Gately et al., 2009), as well as in CHR (Fusar-Poli et al., 2012; Loewy et al., 2016; Seidman et al., 2010). One study (Seidman et al., 2010) reported that verbal memory predicted a more rapid transition to psychosis and hence, appeared related to increased psychotic symptoms. In a study where individuals with CHR were receiving computerized verbal memory training, participants obtained significantly improved positive symptom levels compared to a control group (Loewy et al., 2016).

The finding that better executive function scores were positively correlated with negative symptoms in CHR was surprising and contradicted other studies. Looking to established psychosis, a meta-analysis in schizophrenia showed a robust positive association between negative symptoms and executive deficits (Dibben et al., 2009). However, there are some exceptions where no such association has been demonstrated (Bozikas et al., 2004; Joyce et al., 2002). Lim et al. (2016) found executive functions to be the one cognitive function with the weakest association to negative symptoms. Further, Harvey et al. (2006) have concluded that cognitive deficits and negative symptoms are best explained as separate domains, in line with the conclusion from the NIMH-MATRICS Consensus Statement on negative symptoms (Kirkpatrick et al., 2006). Finally, a small study on higher order cognitive functions such as motivation and metacognition in psychosis and attenuated psychotic symptoms also found no associations with negative symptoms (ten Velden Hegelstad et al., 2020). Our results may serve as an indication that impairments in executive functions are less profound in CHR with negative symptoms compared to patients with schizophrenia with the same symptom pattern. In the investigation of executive functions related to negative symptoms in schizophrenia Bagney et al. (2013) found that the number of preservative errors on the Wisconsin Card Sorting Test (WCST) increased as the illness progressed. Hence, they recommend considering the duration of illness in further research regarding the relationship between executive functions and negative symptoms.

Another explanation may be sought in selection bias, cautioning interpretation of our finding. Negative symptoms were not a part of our inclusion CHR criteria. Thus, our study – as well as other CHR studies that apply the same inclusion criteria- fails to include persons having negative but no attenuated positive symptoms. If future research finds that, in line with another study from our group, that early or attenuated negative symptoms are indicative of a trajectory towards psychosis (Bjornestad et al., 2021), perhaps inclusion criteria should be amended to correct this. Then studies can investigate more closely the association of attenuated negative symptoms with neurocognitive functioning much in the same way as with positive symptoms. This could shed light on mechanisms in developing psychosis.

There is another methodological issue possibly influencing results as well. Measuring negative symptoms is hampered by validity concerns. Their phenomenological description is a conglomerate of factors and could be expressions of depression, fatigue, behavioral aspects of substance use, “having a bad day”, going through puberty, and others. To address whether this was the case in our study, we conducted a secondary analysis to compare how negative symptoms were associated with the other symptom domains. They were strongly associated with general and disorganization, but not with positive symptoms. This indicates that negative symptoms in CHR may be expressions of distress and may simply be markers of psychological phenomena within the range of normal development. In sum, this finding still confronts us with an unanswered question to be investigated in future studies.

When including age and gender as covariates to compare positive- and negative symptoms with cognition these covariates had little to no effect. However, findings indicated a statistically significant negative association between disorganization symptoms and verbal fluency when controlled for age and gender. Impairments in verbal fluency are common in CHR (Fusar-Poli et al., 2012). Becker et al. (2010) suggested these to be among of the earliest impairments in the prodromal phase, and a possible predictor for further psychosis development. It appears intuitive that subjects with disorganized thinking could have trouble producing words fluently and adequately on a test. Further, language is the overt expression of thoughts. Disorganized thinking can often be in violation of the boundaries of most conventional, or coherent, thought. Unusual ideas may contribute to the production of novel answers in the form of words generated in the wrong category during testing, and in ambiguous words. According to Rocca et al. (2018) disorganization is the least studied symptom dimension of schizophrenia. This is also the case for CHR studies, and we recommend future studies to investigate disorganization symptoms and verbal fluency.

Neither of the cognitive composites was associated with symptom levels, except for a trend for positive symptoms. This contradicts studies in psychosis, finding negative symptoms to be related to general cognition (Keefe et al., 2006). One can speculate whether this seeming paradox could be related to illness stage. Of note, individuals with CHR in most cases never develop psychosis; however assuming that they share a common vulnerability with those who do, it is worth considering. Heilbronner et al. (2016) present a summary of how clinical symptoms alter during the stages of psychotic disorders; positive symptoms more likely to remit within 2 years after onset, and negative symptoms to be stabilized after 15. Also, Bagney et al. (2013) argue that investigating negative symptoms and executive functions, the stage of the illness should be taken into consideration. Aase et al. (2021) found a significant reduction in positive symptoms during the first six months in CHR, similar to a study by Barbato et al. (2013). Positive symptoms may also be less prominent in FEP and schizophrenia at later stages due to antipsychotic medication targeting positive symptoms (Correll and Schooler, 2020). In sum, this study yields some interesting and novel findings on the associations between cognitive function and specific symptom domains in CHR. They indicate specific associations between symptoms and cognition. As the study is small, it may serve as giving direction to further investigation.

### 4.1 Strengths and limitations of the study

The major strengths of this study include the population based approach and thorough diagnostic assessments of the participants. The sample is representative of age, gender, symptomatology, and conversion to psychosis when compared to a meta-analysis by Fusar-Poli et al. (2020). Further, the SIPS is regarded as one of the two most valid and reliable instruments to assess CHR (Mam-Lam-Fook et al., 2017). We also consider the investigation of positive-, negative- and disorganization symptoms with the same test battery in the same sample as a strength of our study.

Including cognitive composite scores based on PCA's can be viewed as a strength. Our sample size is small compared to the CATIE and VA studies, and thus we computed two composite scores for the purpose of comparison. Given the limited reliability of PCA performed on small data sets such as ours, we based the decision of a single composite on previous, larger studies (Harvey et al., 2016; Keefe et al., 2006). Even so, the actual weighting of the different tests/domains was estimated from the present data, and this may or may not be in line with other studies. The explorative PCA composed of the five cognitive domains yield one factor accounting for 46% of the test variance, compared to 45% in the CATIE schizophrenia study by Keefe et al. (2006). In a CHR study by Barbato et al. (2013) a cognitive factor was computed by PCA of the individual tests, and 32% of the variance was explained by the first factor. However, in our data the composite score from the single tests explained 29% of the variance. Thus, computing a cognitive composite score in CHR may enhance the robustness of further analysis in the same way as for subjects with schizophrenia.

Finally, a strength is the choice of CVLT as a measurement for verbal memory. In a recent review of measures for verbal memory (Kilciksiz et al., 2020) CVLT was used in 27% of FEP studies, which strengthens the generalizability of the results in the present study.

Employing a self-reported executive problems and essential information from family and informants such as Behavior rating inventory of executive function (BRIEF) (Roth et al., 2005) could have complemented our finding regarding the association between positive symptoms and executive deficits. In the present study however, daily life functioning was estimated using GAF-M as a proxy for level of functioning.

The relatively small size of the sample is a general limitation of our study, and is also reflected in wide confidence intervals for the correlation estimates. Further, the large number of possible associations we assessed increases the risk of false-positive findings, which is a general caveat in exploratory studies. More recently however, researchers have argued that adjusting for multiple comparisons in psychiatry and psychology may have disadvantages regarding Type II errors, resulting in novel findings being overseen (de Carvalho Alves and da Rocha, 2019). American Statistical Association also state that *p* values reported without a context may provide insufficient information (Wasserstein and Lazar, 2016) Therefore, as recommended in the new editorial guidelines for reporting statistical findings in the New England Journal of Medicine (Harrington et al., 2019), we reported point estimates and confidence intervals (95%) for the effect sizes. The statistically significant correlations between cognition and symptoms varied from 0.34 to 0.28, which implies that our correlations are moderate to small (Cohen, 1988). However, according to a paper published in Nature by Kapur et al. (2012) effect sizes are small to medium in most studies within biological psychiatry, and the authors indicate that this is also the case in other fields of psychiatry. Since, to our knowledge, this study is the first to elucidate the relationship between cognition and all three symptoms domains in addition to apply a general cognitive composite score, the findings from the present study may still add to the knowledge in the field, providing hypotheses for future confirmatory research.

### 4.2 Conclusions

In this study, associations between positive symptoms in CHR and impairments in executive functions were in line with expectations. Contrary to studies in psychosis we found higher negative symptom levels to be associated with better executive performance. This finding may indicate that executive function is less tightly associated with negative symptoms in CHR compared to schizophrenia, perhaps because in CHR, they represent a conglomerate of several phenomena not all related to psychopathology. A cognitive composite, as opposed to domain-specific cognitive scores, was not associated with any symptom dimension, except for a trend possibly indicating some association with attenuated positive symptoms. Our results may indicate that both type and strength of associations between cognitive functions and symptoms differ across illness stages and symptom severity.

## CRediT authorship contribution statement

IA and JHL wrote the first drafts for the paper. IA, ID and JHL conducted the statistical analyses. IA, JHL, and WtVH interpreted the results and wrote the second draft of the paper. JOJ and IJ outlined the overall Primary Prevention of Psychosis (POP) study. All the authors provided detailed comments on the paper across several drafts and contributed in editing the final manuscript, also they were available for input during the process.

## Ethics

The study was approved by the local Institutional Review Board Regional Committee for Medical Research Ethics Sør-Øst (ref. no. 2009/949). Parents or legal guardians gave informed consent for individuals younger than 16 years of age, as in Norway, individuals are legally able to consent without parental approval from the age of 16. The individuals were offered treatment in clinical mental health services at Stavanger University Hospital during a 24 month follow-up period. The present study was conducted according to the requirements of the Declaration of Helsinki/Code of Ethics of the World Medical Association (2013).

## Funding

The present study received support by way of a personal grant from Health West Foundation to IA 13968. Two grants from Health West Foundation 911508 and, later on 911881. It was also supported by way of a grant 913184 from the 10.13039/100009471Norwegian Extra Foundation for Health and Rehabilitation through EXTRA funds.

## Declaration of competing interest

The authors report no conflict of interest.

## Appendix A Supplementary data

Supplementary tables S1_Principal component analysis. Image 1

S2_Scatterplots Image 2
