# Supplementary material for: Associations between symptom and neurocognitive dimensions in clinical high risk for psychosis
Source: Schizophr Res Cogn. 2022 Jun 2;29:100260. doi: 10.1016/j.scog.2022.100260 (PMC9168614; doi:10.1016/j.scog.2022.100260)
Supplement: Supplementary file 1 — Supplementary tables S1_Principal component analysis. [file mmc1.docx]

Table S1: Results from principal component analysis of the 15 individual cognitive tests

| **COGNITIVE DOMAIN** | **NAME OF TEST** | **LOADING FIRST PC** |
| --- | --- | --- |
|  |  |  |
| ***Attention*** | D-KEFS CWIT Color Naming* | 0.23 |
|  | D-KEFS CWIT Word Reading* | 0.17 |
|  | WAIS-III Digit Span Forward | 0.28 |
|  |  |  |
| ***Verbal memory*** | CVLT-II List A Total Recall | 0.29 |
|  | CVLT-II Short-Delay Free Recall: List A | 0.27 |
|  |  |  |
| ***Verbal fluency*** | D-KEFS VFT Letter Fluency | 0.29 |
|  | D-KEFS VFT Category Fluency (animals) | 0.29 |
|  | D-KEFS VFT Category Fluency (names) | 0.12 |
|  | D-KEFS VFT Category Switching | 0.25 |
|  |  |  |
| ***Executive functions*** | WAIS-III Digit Span Backward ***(Working Memory)*** | 0.35 |
|  | D-KEFS CWIT Inhibition* ***(Inhibition)*** | 0.20 |
|  | TMT-B* (***Cognitive Flexibility)*** | 0.30 |
|  | D-KEFS CWIT Inhibition/Switching**** (Cognitive flexibility)*** | 0.15 |
|  |  |  |
| ***General intelligence*** | WAIS-III Vocabulary | 0.21 |
|  | WAIS-III Block Design | 0.34 |
|  |  |  |

Principal component analysis was based on the correlation matrix of the test z-scores obtained by comparison with a group of normal controls. Tests for which a higher score indicates poorer performance (marked with *) were reversed before calculating z-scores. The first principal component explained 29% of the variance in the individual standardised test scores. PC=Principal Component, D-KEFS CWIT = Delis-Kaplan Executive Function System Color Word Interference Test (“Stroop”), D-KEFS VFT = Delis-Kaplan Executive Function System Verbal Fluency Test (D. Delis, Kaplan, & Kramer, 2001), WAIS-III = Wechsler Adult Intellegence Scale (Wechsler, 1997), WMS-III =TMT = Trail Making Test (War Department Adjutant General`s Office, 1944), CVLT-II = California Verbal Learning Test (D. C. Delis, 2000)

Table S2: Results from principal component analysis of the five cognitive domains

| **COGNITIVE DOMAIN** | **LOADING FIRST PC** |
| --- | --- |
|  |  |
| Attention | 0.47 |
| Verbal memory | 0.32 |
| Verbal fluency | 0.39 |
| Executive functions | 0.53 |
| General intelligence | 0.50 |
|  |  |

Principal component analysis was based on the correlation matrix of the domain scores, The first principal component explained 46% of the variance in the individual domain standardised scores. PC=Principal Component,
