# Supplementary figures and images for: Associations between symptom and neurocognitive dimensions in clinical high risk for psychosis
Source: Schizophr Res Cogn. 2022 Jun 2;29:100260. doi: 10.1016/j.scog.2022.100260 (PMC9168614; doi:10.1016/j.scog.2022.100260)

S2 Scatterplotts


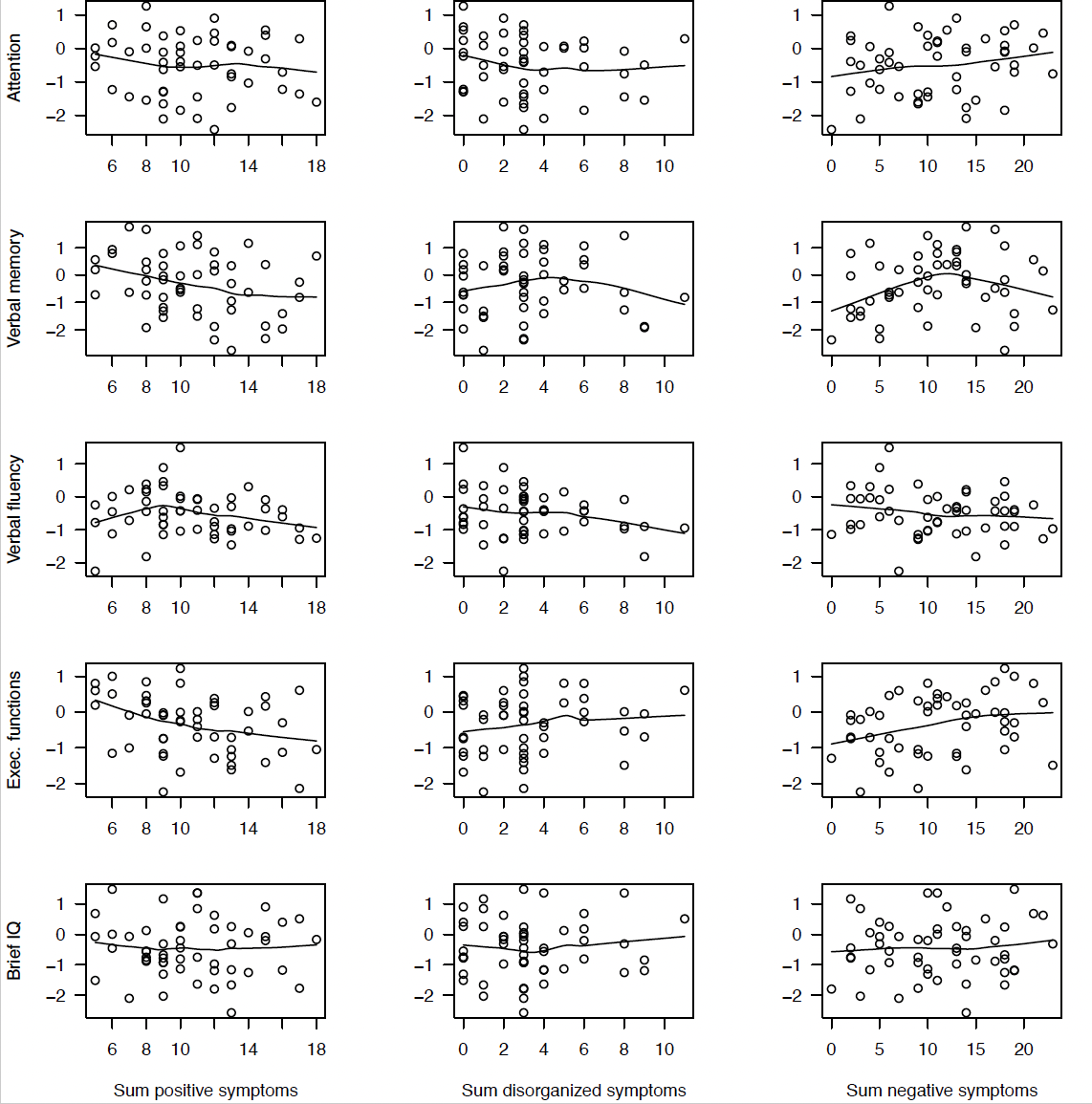

Supplement: Supplementary file 2 — S2_Scatterplots [file mmc2.docx]
